# Attitudes toward Vaccinations and Vaccination Coverage Rates among Dental Students in Greece

**DOI:** 10.3390/ijerph19052879

**Published:** 2022-03-01

**Authors:** Helena C. Maltezou, Christos Rahiotis, Maria Tseroni, Phoebus Madianos, Ioannis Tzoutzas

**Affiliations:** 1 Directorate of Research, Studies and Documentation, National Public Health Organization, 15123 Athens, Greece; 2 School of Dentistry, National and Kapodistrian University of Athens, 11527 Athens, Greece; craxioti@dent.uoa.gr (C.R.); pmadian@dent.uoa.gr (P.M.); tzoudent@dent.uoa.gr (I.T.); 3 Directorate of Epidemiological Surveillance of Infectious Diseases, National Public Health Organization, 15123 Athens, Greece; mariatseroni@gmail.com

**Keywords:** vaccination, immunization, coverage, dental school, dental students, immunity, susceptibility, mandatory vaccination

## Abstract

Our aim was to study attitudes toward vaccinations, full vaccination rates and susceptibility rates against vaccine-preventable diseases among students attending a University Dental School. A total of 134 students were studied. Full vaccination rates were as follows: 56.5% against measles and mumps, 70.6% against rubella, 32.3% against varicella, 44.1% against hepatitis A, 45.9% against hepatitis B, and 87.7% against COVID-19. In the past decade, 63.2% of students had received a booster shot against tetanus–diphtheria, 47.8% against pertussis, and 28.1% against poliomyelitis, while 29.4% of students had been vaccinated against influenza in the past year. Susceptibility rates were 40.4% for measles, 42.4% for mumps, 28.3% for rubella, 32.3% for varicella, 55.3% for hepatitis A, 54.1% for hepatitis B, 36.8% for tetanus–diphtheria, 52.2% for pertussis, and 71.9% for poliomyelitis. Overall, 123 (91.8%) students favored mandatory vaccinations, mainly for all dentists (88.4%), while 11.6% of students favored them only for dentists who provide care to high-risk patients. In conclusion, most dental students favored mandatory vaccinations, yet we found significant vaccination gaps and susceptibility rates against vaccine-preventable diseases. Vaccinations for dental students should be intensified. A national vaccination registry for healthcare personnel including dental students is urgently needed.

## 1. Introduction

Despite the wide implementation of routine vaccination programs in the past decades, several vaccine-preventable diseases (VPDs) are still transmitted in the context of the provision of healthcare services [1]. Unvaccinated healthcare personnel (HCP) are at increased risk for occupational acquisition of VPDs and subsequent transmission to susceptible patients, with significant associated morbidity and mortality [2,3,4,5,6]. Currently, vaccinations of HCP are considered a key infection-control measure to directly protect them, but also to indirectly protect vulnerable patients through herd immunity [7,8]. Indirect protection is of the utmost importance for patients who do not elicit sufficient immunity after vaccination or for whom vaccination is contraindicated (e.g., immunosuppressed patients, elderly, and pregnant women) [1]. Healthcare students, including dental students, are often missed by infection control and occupational medicine systems because of their shared time between clinical practice and lecture halls [9]. Nonetheless, healthcare students should comply with the same vaccination recommendations and infection prevention measures as HCP [9]. 

As dental HCP, dental students constitute a high-risk group for several VPDs including coronavirus disease 2019 (COVID-19), but also cross-infection, because of particular conditions that are frequently encountered in dental clinics during the provision of dental services. These include proximity with the oral cavity and the upper respiratory system, droplets of secretions, saliva, and blood of patients, invasive procedures, and the use of instruments that produce aerosols [6,10,11,12,13,14,15]. These can cause the transmission of infection between dental students, dental practitioners, and patients. Studies indicate that dental students are prone to needle-stick injuries and percutaneous and mucous membrane exposures [14,16]. Therefore, to address occupational health needs in dental settings, effective infection control is required, including adequate vaccinations of dental HCP [17,18]. 

In 2012–2013, we conducted a cross-sectional survey among healthcare students in Athens, which showed vaccination gaps and significant susceptibility rates against measles, mumps rubella, varicella, hepatitis A, hepatitis B, and tetanus–diphtheria [19]. 

The aim of the current cross-sectional survey was to estimate full vaccination coverage rates and susceptibility rates against VPDs among dental students of the University of Athens, Greece as of 2021. 

## 2. Material and Methods

### 2.1. Study Design and Setting 

This is a cross-sectional survey. The Administration of the Dental School of the University of Athens was invited and agreed to participate in the study. The survey was conducted from 22 November through 19 December 2021.There are 600 undergraduate students in the Dental School. 

### 2.2. Data Collection

A structured questionnaire was developed based on the questionnaire used in our 2012–2013 survey [19]. The questionnaire was accessible through a web-based link and was distributed to all undergraduate dental students via an inviting email sent from the Administration Office of the Dental School. The following data were collected: demographic characteristics, history of natural infection and vaccination history (including number of doses) against measles, mumps, rubella, varicella, hepatitis A, hepatitis B, tetanus–diphtheria, pertussis, poliomyelitis, seasonal influenza, and COVID-19. Information about the date of last vaccination against tetanus–diphtheria, pertussis and poliomyelitis, influenza vaccination in the past (2020–2021) season, and their intention to have aninfluenza vaccination in 2021–2022 season was also collected. The attitudes of students toward mandatory vaccinations were also recorded, and personalized scenarios about vaccinations were used. Students were advised to consult their Pediatric Vaccination Booklet for vaccinations and history of natural infection, if available. Data were self-reported and anonymous. 

### 2.3. Definitions

Full vaccination was defined as one dose for rubella, two doses for measles, mumps, varicella, hepatitis A, and COVID-19 (the mRNA COVID-19 vaccines were administered to HCP), and three doses for hepatitis B, delivered within the appropriate time scheme for each VPD. Full vaccination against tetanus–diphtheria, pertussis, and poliomyelitis was defined as one booster dose containing the respective antigen(s) within the past 10 years. Full vaccination against influenza was defined as one dose of influenza vaccine in the past season. 

Self-reported immunity against VPDs was defined as either a history of full, up-to-date vaccination and/or a history of past infection leaving permanent immunity. Past infection was not considered for tetanus–diphtheria, and pertussis because of waning immunity after natural infection. History of hepatitis B was also not considered for immunity since acute infection can progress directly to chronic infection. Susceptibility to VPD was defined as a lack of self-reported immunity, and was estimated as follows: (1 − immunity) × 100%. Susceptibility was not estimated for influenza and COVID-19 given the need for repeated vaccinations.

### 2.4. Ethical Issues 

The participation of students was voluntary and anonymous. The study was approved by the Ethics Committee of the Dental School (approval code: 490). The data were managed in accordance with the national and European laws.

### 2.5. Statistical Analysis

Proportions were calculated using the non-missing values as denominators. Logistic regression analysis was used to identify factors associated with vaccination status, susceptibility status against VPDs, and attitudes towards mandatory vaccinations for dentists. The odds ratio (OR) and confidence interval (CI) were estimated. *p*-values of ≤0.05 were considered statistically significant. The statistical analysis was carried out using STATA software (Stata Corporation, College Station, TX, USA, version 17).

## 3. Results

A total of 134 dental students (92 women; 68.7%) participated in the survey (response rate: 22.3%). Their mean age was 21.4 (range: 18–38) years. Regarding the academic year, there were 31 (23.1%) first-year students, 38 (28.4%) second-year students, 16 (11.9%) third-year students, 23 (17.2%) fourth-year students, and 26 (19.4%) fifth-year students. Comorbidities were reported by 5 (3.7%) students.

Table 1 shows full vaccination rates of dental students. The highest full vaccination rate was found against COVID-19 (87.7%), rubella (70.6%), measles and mumps (56.5% each), and hepatitis B (45.9%). Overall, 84% of students had been vaccinated in the past against tetanus–diphtheria (mean number of 3.5 doses; range: 1–7 doses), 72.7% against pertussis (mean number of 3 doses; range: 1–7 doses), and 80.3% against poliomyelitis (mean number of 3.75 doses; range: 1–7 doses). However, only 63.2%, 47.8%, and 28.1% of students who provided an answer had received a booster shot against tetanus–diphtheria, pertussis and/or poliomyelitis in the past ten years, respectively. Finally, 49 (37.1%) out of 132 students who answered this question stated their intention to have aninfluenza vaccine in the upcoming (2021–2022) influenza season. Logistic regression analysis showed a significant association between being a female and having a history of two doses of measles–mumps–rubella (MMR) vaccine (OR: 5.06, 95% CI: 1.88–13.60, *p*-value = 0.001) or being fully vaccinated against hepatitis B (OR: 2.88, 95% CI: 1.16–7.16, *p*-value = 0.023). No significant association was found between any full vaccination against other VPDS and age or academic year of students (data not shown). 

Regarding natural immunity, a history of varicella was reported by 44 (33.6%) students, a history of measles by 4 (3%) students, and a history of mumps, rubella, pertussis, hepatitis A, and hepatitis B by 1 (0.7%) student. Susceptibility rates of dental students were estimated as follows: 40.4% for measles, 42.4% for mumps, 28.3% for rubella, 32.3% for varicella, 55.3% for hepatitis A, 54.1% for hepatitis B, 36.8% for tetanus–diphtheria, 52.2% for pertussis, and 71.9% for poliomyelitis (Table 2). Logistic regression analysis showed that compared with male students, female students were less likely to be susceptible against measles (OR: 0.24, 95% CI: 0.95–0.62, *p*-value = 0.003) and varicella (OR: 0.33, 95% CI: 0.13–0.80, *p*-value = 0.015). No association was found between susceptibility rates against other VPDs and age or academic year of students (data not shown). 

Regarding the attitudes of dental students toward vaccinations, 123 (91.8%) students stated that vaccinations should be mandatory for dentists. Similarly, using personalized scenarios, 82.9% to 89.8% of students favored mandatory vaccinations for HCP (Table 3).

Of the 123 students who favored mandatory vaccinations for dentists, 92 (75.4%) students stated that all vaccinations should be mandatory for dentists while the remaining 31 (24.6%) favored mandatory vaccinations against particular VPDs. Among the latter, the highest acceptance rates of mandatory vaccination concerned hepatitis B and hepatitis A vaccines (85.7% and 67.9%, respectively), followed by MMR, COVID-19, and tetanus–diphtheria vaccines (57.1%, 57.1%, and 53.6%, respectively). Mandatory vaccination against influenza was accepted by 28.6% of students. Finally, of the 121 students who answered this question, 107 (88.4%) stated that vaccinations should be mandatory for all dentists while 14 (11.6%) believe that vaccinations should be mandatory only for dentists who provide dental care to high-risk patients. Logistic regression analysis found no association between acceptance rate of mandatory vaccinations, gender, age, and academic year of students (data not shown). However, students who had received a booster dose of tetanus–diphtheria or pertussis in the past decade were less likely to support mandatory vaccinations compared to students who have not received a booster shot against tetanus–diphtheria (OR: 0.61; 95% CI: 0.61, CI: 0.50–0.74, *p*-value < 0.001) or pertussis (OR: 0.45, 95% CI: 0.33–0.63, *p*-value < 0.001, respectively). No other association was found between full vaccination status and acceptance of mandatory vaccinations. In addition, no association was found between full vaccination against any VPD and intention to have aninfluenza vaccine in the upcoming season (data not shown).

## 4. Discussion

We conducted a cross-sectional survey to estimate full vaccination rates and susceptibility rates against VPDs among dental students of the University of Athens. To the best of our knowledge, the published data are scarce among dental students. As in most countries, there is no centralized vaccination registry in Greece to follow vaccination rates among HCP personnel, including dental students. In our study, the overwhelming majority of participating students favored mandatory vaccinations for dentists. However, significant vaccination gaps and susceptibility rates were found against almost all VPDs. The modified epidemiology of several VPDs in the past two decades in many developed countries accounts largely for that. The high full vaccination rate against COVID-19 is largely attributed to the implementation of mandatory vaccination policy for healthcare students in clinical practice. Our findings should trigger the implementation of vaccination programs, specifically for healthcare students.

In our study, most dental students have been fully vaccinated against COVID-19. COVID-19 represents an occupational risk for dentists [10,20,21]. Vaccination against COVID-19 significantly reduces morbidity and absenteeism among HCP [22]. However, in many settings, HCP have been hesitant to have a COVID-19 vaccine. A recent survey among 6639 dental students (mean age: 22.1 years, 70.5% females) from 22 countries globally found an overall 22.5% rate of COVID-19 vaccine hesitancy, which was more pronounced in low- and lower-middle income countries [23]. A survey among 340 Greek HCP found an overall 78.5% level of acceptance of COVID-19 vaccination, which was significantly higher (82.5%) among dentists [24]. Starting in September 2021, Greece made COVID-19 vaccination mandatory for all HCP, including healthcare students, while non-compliant students are excluded from clinical practice [25]. As of 1 January 2022, 93% of practicing dentists in Greece have been fully vaccinated against COVID-19 (Regional Dental Societies, personal communication).

Our study showed suboptimal vaccination rates against all other VPDs. In particular, less than half of dental students who could recall the entire number of vaccine doses have been fully vaccinated against hepatitis B. In contrast, in another study we conducted in early 2020, 96.5% of 385 military recruits (mean age: 23.5 years) had been fully vaccinated against hepatitis B [26]. In our study, the low full vaccination rate against hepatitis B is attributed to the large number of participating freshmen and sophomores. On the contrary, the third-, fourth-, and fifth-year students are mandatorily vaccinated against hepatitis B as a prerequisite of the Infection Control Committee to participate in clinical activities [27]. Exposure incidents occur frequently in dental settings and hepatitis B has been well-recognized as an occupational risk for dentists [12]. A survey conducted a decade ago at the University of Frankfurt among 153 dental students and 112 dental healthcare personnel revealed that dental students had nearly twice the number of needle-stick injuries compared with dentists with more or less than 10 years of working experience [14]. Insufficient use of personal protective equipment was also frequent [14]. However, the past three decades occupational infections of HCP have decreased substantially due to the hepatitis B vaccination but also due to implementation of preventive measures, including the evaluation and management of incidents of blood exposure [12,13,20]. University-based studies across the world indicate that the percentage of dental students who have completed their vaccination schedule against hepatitis B ranges from 9.4% to 97%, often in association with gaps in their knowledge and practices about blood-borne infections [28,29,30,31,32,33,34,35].

In our study, only 56.5% of surveyed dental students had received two doses of MMR vaccine, despite the fact that the respective recommendations have been issued more than three decades ago and despite the large measles epidemic that occurred in Greece in 2017–2018 [3]. In addition, female students had significantly higher full vaccination rates with MMR and hepatitis B vaccines. Such differences between sexes can be attributed to differences in knowledge or risk perception about VPDs and vaccines and/or differences in compliance rates with vaccination recommendations. Significantly less than half of the surveyed students have been fully vaccinated against hepatitis A and varicella, which is partially attributed to the fact that hepatitis A and varicella vaccines were included in the Greek routine pediatric vaccination program in 2007. Overall, full vaccination rates in our students lagged behind those found in our 2013 study among 150 healthcare students (mean age: 22.3 years) [19] as well as those in another study of ours conducted among 385 military recruits (mean age: 23.5 years) in early 2020 [26]. In the 2013 study among healthcare students, full vaccination rates were as follows: 68.5% for measles and mumps, 79.5% for rubella, 19.6% for varicella, 46.3% for hepatitis A, 70% for hepatitis B, and 80.25 for tetanus–diphtheria [19]. 

Moreover, in our study, only 63.2% of dental students had received a booster shot against tetanus–diphtheria, and even fewer had received a pertussis-containing shot or a poliomyelitis-containing shot in the past decade. Similarly, a 2013 survey across 21 states in the United States of America found an overall 47.2% self-reported vaccination rate with tetanus, diphtheria, and acellular pertussis (Tdap) vaccine, which was higher among physicians (66.8%) compared with 39.3% in dentist offices [36]. Finally, as reported by others (31.6%) [37], in our study, only 29.4% of students had been vaccinated against influenza the past year. Educational campaigns on the expected benefits of HCP influenza vaccination for them and their patients are imperative [38].

A worrying finding of our study was that a significant percentage of students were susceptible to all studied VPDs. In particular, approximately four out of ten students were susceptible against measles or mumps, one out of four students was susceptible against rubella, and one out of three students was susceptible against varicella. These findings reflect the mixture of suboptimal full vaccination rates and the modified epidemiology of many VPDs in the post-vaccine era [39]. Indeed, except varicella, very few dental students had a history of natural measles, mumps, or rubella. In contrast, lower susceptibility rates were found in a study among 150 healthcare students conducted in Athens a decade ago (20.5% for measles, 26.4% for mumps, 13.9% for rubella, and 15.7% for varicella) [19]. The increased susceptibility rates found in our study compared to the study conducted among healthcare students ten years ago are of concern, given that most VPDs are associated with serious morbidity and increased mortality during adulthood. There is an urgent need to identify susceptible healthcare students and raise vaccination rates [1]. Organization and practical issues, including on-site vaccination, vaccination registries, follow-up procedures and reminder systems, should also be addressed [38]. Undergraduate and postgraduate programs of dentistry schools should also provide education about VPDs, increase awareness about vaccines and therefore influence vaccination decision-making and raise vaccination rates [18]. Overall, vaccination programs for healthcare students, including dental students, should be developed.

Another finding of our study was that mandatory vaccinations for all dentists were supported by most dental students. Such a policy can indirectly protect all patients, regardless of their susceptibility status. In addition, three out of four supporters of mandatory vaccinations stated that mandatory vaccination policies should concern all vaccines. However, a significant number of students supported mandatory vaccination policies mostly against hepatitis B and hepatitis A only. The perception of the risk of morbidity but mainly mortality of VPDs influences vaccination decision-making [40]. 

The current survey was conducted in the largest Dental School of Greece, which gave us the opportunity to study a significant number of dental students of all academic years. The response rate of 22.3% is within the range (15% to 36.4%) of other similar studies conducted in Greece in the past decade [19,24,41]. Recall bias is a potential limitation. However, previous studies among healthcare students in Germany and Italy indicate good correlation between self-reported immunity and serologic findings against several VPDs [42,43]. Differences in attitudes between dental students with clinical experience and newly admitted students may occur [44] and are also a potential limitation.

## 5. Conclusions 

This is one of few studies conducted to estimate vaccination rates among dental students globally. The overwhelming majority of participating students favored mandatory vaccinations for dentists. However, significant vaccination gaps and susceptibility rates were found against almost all VPDs. The modified epidemiology of several VPDs in the past two decades in many developed countries accounts largely for that. The high full vaccination rate against COVID-19 is largely attributed to the implementation of mandatory vaccination policy for healthcare students in clinical practice. Our findings should trigger the implementation of vaccination programs specifically for healthcare students, including dental students in site. A national registry to follow vaccination coverage rates of HCP including dental students in real time is imperative.

## Figures and Tables

**Table 1:** Full vaccination rates of dental students.

VPD	Full Vaccination Rates (%)
Measles (*n*= 92)	56.5
Mumps (*n* = 92)	56.5
Rubella (*n* = 92)	70.6
Varicella (*n* = 96)	32.3
Hepatitis A (*n* = 93)	44.1
Hepatitis B (*n* = 109)	45.9
Tetanus–diphtheria ^1^ (*n* = 68)	63.2
Pertussis ^1^(*n* = 46)	47.8
Poliomyelitis ^1^ (*n* = 57)	28.1
Seasonal influenza ^2^ (*n* = 129)	29.4
COVID-19 (*n* = 130)	87.7

*n*: number of students who answered the question about the respective vaccine doses. VPD: vaccine-preventable disease; COVID-19: coronavirus disease 2019. ^1^ Booster dose the past 10 years. ^2^ Influenza vaccination in the 2020–2021 season.

**Table 2:** Susceptibility rates against VPDs of dental students.

VPD	Susceptibility Rates (%)
Measles (*n* = 94)	40.4
Mumps (*n* = 92)	42.4
Rubella (*n* = 92)	28.3
Varicella (*n* = 102)	32.3
Hepatitis A (*n* = 94)	55.3
Hepatitis B (*n* = 109)	54.1
Tetanus–diphtheria (*n* = 68)	36.8
Pertussis (*n* = 46)	52.2
Poliomyelitis (*n* = 57)	71.9

*n*: number of students who answered the question about the respective vaccine doses. VPD: vaccine-preventable disease.

**Table 3:** Attitudes of dental students regarding mandatory vaccinations using personalized scenarios.

Question	Agreement Rate
1. If a member of your family is immunocompromised, should the HCP caring for him/her be immune against measles? (*n* = 129)	82.9%
2. If your newborn baby is admitted in a NICU, should the HCP caring for it be immune against varicella? (*n* = 128)	89.8%
3. If a member of your family has COPD, should the HCP caring for him/her get influenza vaccine every year? (*n* = 131)	86.2%

HCP: healthcare personnel; NICU: neonatal intensive care unit; COPD: chronic obstructive disease.

## Data Availability

Not applicable.
